# Eldecalcitol prevents muscle loss by suppressing PI3K/AKT/FOXOs pathway in orchiectomized mice

**DOI:** 10.3389/fphar.2022.1018480

**Published:** 2022-10-28

**Authors:** Haichao Zhang, Zheng Ke, Shuangshuang Dong, Yanping Du, Wenjing Tang, Minmin Chen, Weijia Yu, Qun Cheng

**Affiliations:** ^1^ Department of Osteoporosis and Bone Disease, Research Section of Geriatric Metabolic Bone Disease, Shanghai Geriatric Institute, Huadong Hospital Affiliated to Fudan University, Shanghai, China; ^2^ Medical Division, Chugai Pharma China Co., Ltd, Shanghai, China

**Keywords:** bone strength, eldecalcitol, muscle atrophy, ORX, PI3K/AKT/FOXOs

## Abstract

Elderly male patients are susceptible to develop osteoporosis and sarcopenia, especially those with fragility fractures, hypogonadism, and prostate cancer with androgen deprivation therapy. However, at present, very few treatments are available for men with sarcopenia. Previous preclinical studies in ovariectomized rats have shown the promising effects of eldecalcitol in ameliorating the bone strength and muscle atrophy. We thus investigated the effects of eldecalcitol on androgen-deficient male mice. Six-week-old male mice underwent orchiectomy (ORX) or sham surgery. Mice were randomly divided into 4 groups (*n* = 12/per group), including 1) sham mice, 2) ORX group, 3) ORX eldecalcitol 30 ng/kg, and 4) ORX eldecalcitol 50 ng/kg. Eldecalcitol increased bone mass and strength of femur in ORX mice. Eldecalcitol 30 ng/kg dose completely rescued ORX-induced muscle weakness. The RT-qPCR showed that eldecalcitol enhanced the mRNA levels of type I and IIa fibers. The expression levels of MuRF1 and Atrogin-1 of gastrocnemius in the eldecalcitol groups were much lower than that of the ORX group. It is assumed that eldecalcitol potentially acts *via* PI3K/AKT/FOXOs signaling pathway. These findings provide evidence for evaluating eldecalcitol as an investigational treatment for male patients with sarcopenia and osteoporosis.

## Introduction

Osteoporosis and sarcopenia are diseases that affect the skeletal muscle system and are highly prevalent among the elderly people (Wang et al., 2015). Sarcopenia is characterized by an age-related loss of skeletal muscle mass and strength, with decline in hormone levels and number of neuromuscular junctions, increased inflammation, and decline in the physical activity or inadequate nutrition (Santilli et al., 2014). Osteoporosis is a progressive bone disease that causes weak and brittle bones, a decrease in bone mass and density leading to debilitating fragility fractures (Zeng et al., 2019; Zheng and Luo, 2020). Both these diseases also reduce the quality of life and mobility, resulting in significant morbidity and mortality (Noguchi et al., 2013).

Osteoporosis causes an estimated 8.9 million fractures worldwide annually in 20%–25% of men (Zheng and Luo, 2020; The Lancet Diabetes and Endocrinology, 2021). It is estimated that China alone has 13% prevalence of osteoporosis, and the annual number of osteoporosis-associated fractures is predicted to grow by 4.8 million by 2035 (Zeng et al., 2019). Also, the prevalence of osteoporosis is lower in men than that in women (Lau et al., 2005); however, the rates of sarcopenia are higher in elderly men than that in women in China (Cheng et al., 2014). In addition to old age, the men with fragility fracture, hypogonadism, or prostate cancer with androgen deprivation therapy (ADT) are more susceptible to develop sarcopenia and osteoporosis (Adler, 2014). However, the bone and muscle cells both have androgen receptors, and the ADT causes risk for loss of bone and muscle strength and declined functionality with long-term frailty (Adler, 2014).

The phosphatidylinositol 3-kinase (PI3K) pathway has central roles in protein synthesis, metabolism, and cellular proliferation (Latres et al., 2005). Extensive studies indicated that decreased activity of the PI3K pathway can contribute to muscle atrophy (Bodine et al., 2001). Activating PI3K/AKT signaling regulates skeletal muscle mass and metabolism (Bodine et al., 2001). A major downstream target of PI3K is the serine/threonine kinase AKT (Cantley, 2002). Once AKT is activated, AKT phosphorylates an ever-increaseing list of substrates, including proteins that inhibit apoptosis, induce protein synthesis, proliferation and gene transcription (Glass, 2003). Further, AKT inhibits the FOXO (Forkhead box) family of transcription factors, which control the expression of E3 ubiquitin ligase (Sandri et al., 2004). We speculated that PI3K/AKT/FOXOs pathway is an important target for the treatment of muscle atrophy.

Eldecalcitol (1α,25 [OH]2-2b-(3-hydroxypropyloxy)vitamin D3), an active form of vitamin D, has been widely used for the treatment of osteoporosis (Hagino, 2013; Noguchi et al., 2013; Tao et al., 2016; Matsumoto and Takahashi, 2018). Eldecalcitol enhances the intestinal calcium absorption by vitamin D receptor. Previous studies have demonstrated that the administration of eldecalcitol suppressed bone turnover and increased bone mineral density (BMD) in patients with osteoporosis (Uchiyama et al., 2002; Sakai et al., 2012). A phase 3 controlled study was conducted in patients with osteoporosis that demonstrated better effect with eldecalcitol in increasing BMD and reducing the incidence of bone fractures as compared with alfacalcidol (Matsumoto et al., 2010). In addition to this, various preclinical studies in ovariectomized rats and clinical studies have shown the promising effects of eldecalcitol (Hagino, 2013; Matsumoto and Takahashi, 2018; Wang et al., 2019).

At present, very few treatments are available for sarcopenia in men. Also, there are very few reliable evidences researching the therapeutic effects of viable drugs in men that delay the progression of sarcopenia, and no medications can be affirmatively recommended as treatments for sarcopenia (Ali and Garcia, 2014; Souza, 2021). The pathogenesis of male osteoporosis is also not fully elucidated. Reduced BMD is often correlated with reduced blood testosterone levels, and patients who undergo ADT for prostate cancer treatment showed reduced bone mass. A preclinical study demonstrated the promising effect of eldecalcitol with selective estrogen receptor modulators in complete inhibition of orchiectomy (ORX)-induced, testosterone-depleted bone loss in male mice (Sato et al., 2017). A few clinical studies in Japan have demonstrated the safety and effectiveness of eldecalcitol in male patients with osteoporosis (Noguchi et al., 2013; Kondo et al., 2019).

In addition to increase in bone strength, eldecalcitol has shown positive effects on muscle function in various animal models. For instance, eldecalcitol promoted formation of myogenic cells in a glucocorticoid-treated rats (Kinoshita et al., 2016). Another study reported an anabolic effect of eldecalcitol on the formation of fast-type myosin heavy chain in myogenic cells (Saito et al., 2017). Therefore, in this study, we have investigated the effects of eldecalcitol on androgen deficiency-induced muscle and bone loss along with their possible molecular mechanism in male mice that underwent ORX.

## Materials and methods

### Chemicals and reagents

Medium-chain triglyceride (MCT) was obtained from Shanghai Yuanye Bio-Technology Co., Ltd. Eldecalcitol was provided by Chugai Pharmaceutical Co., Ltd., Tokyo, Japan. Isoflurane was purchased from Abcam Plc. Radioimmunoprecipitation assay buffer was purchased from Cell Signaling Technology, Danvers, MA, United States. Hematoxylin and Eosin were purchased from Beyotime Biotechnology, Nanjing, China. Bicinchoninic acid assay kit was purchased from Pierce, Rockford, IL, United States.

### Animals

All studies involving mice were approved by the Animal Ethics Committee of Fudan University, number (202104060S). C57BL/6J mice were purchased from Hangzhou Ziyuan Laboratory Animal Technology Co., Ltd., China. All the mice were acclimatized and maintained at constant room temperature (RT; 25–27°C), humidity 55%–70%, and 12 h/12 h dark/light cycle, with feed and water *ad libitum* as per Animal Welfare Act regulations and the Guide for the Care and Use of Laboratory Animals (2022) (National Research Council (US) Committee for the Update of the Guide for the Care and Use of Laboratory Animals, 2022).

### Animal study design

Male mice aged 6-week and weighing between 22.1 and 24.8 g were randomly divided into 4 groups with each group containing 12 mice (*n* = 12). After the mice were anesthetized with 2% isoflurane, the mice were divided into 4 groups: 1) Sham group of mice were sham-operated (Sham), 2) ORX group, 3) ORX eldecalcitol 30 ng/kg, and 4) ORX eldecalcitol 50 ng/kg were orchiectomized as described previously (Nastiuk et al., 2007; Davis et al., 2011). Briefly, mice were exposed to 2% isoflurane inhalation to anesthetize before surgery. Mice from the groups (2), (3), and (4) were castrated *via* the scrotal incision. Subsequently, the testicles were removed along with vas deferens and fat pad. Following this, the blood vessels, as well as vas deferens were ligated, and the incision was closed with surgical silk. In the sham group, only the fat pad next to the testicles was removed.

Care was taken to minimize trauma at every step of the procedure (the mice were allowed to recover for 1 week). The sham group 1) and the ORX group 2) were given the MCT vehicle, the ORX eldecalcitol 30 ng/kg group 3) was treated with eldecalcitol at 30 ng/kg, and the ORX eldecalcitol 50 ng/kg group 4) was treated with eldecalcitol at 50 ng/kg by oral gavage, 3 times a week for 12 weeks after surgery. Body weight was measured every week, and the last weight measurement was used for statistical analyses. At the end of the 12-week treatment, the mice were euthanized with an excess of isoflurane and dissected to collect the soleus muscles, gastrocnemius muscles, femur, and tibia for further analysis.

### Measurement of grip strength

The measurement of grip strength was carried out for all groups of animals by using a metal mesh attached to a grip strength meter (KW-ZL Grip Strength Meter, KEW BASIS Nanjing, China) 12 weeks after the surgery. The mice were allowed to grasp the metal mesh attachment by their forelimbs. Once the mice grasped the metal mesh, it was pulled approximately at a rate of 2 cm/s continuously until the mice left the grip (Kitajima and Ono, 2016). The maximum force exerted by the grip was recorded. For each mouse, 10 successive measurements were carried out to replicate data, and the maximum values were used for data analysis to derive muscle force.

### Quantitative computed tomography

At the end of the 12-week treatment, the mice were dissected to obtain femurs in addition to other tissues. The femurs were fixed in 4% paraformaldehyde for 12 h and transferred to 75% ethanol at 4°C before assessing the bone microarchitecture. The femurs were scanned by micro-computed tomography (µCT), and the scanning parameters were in the resolution of 8.96 μm, voltage of 50 kV, and current of 450 mA. The relative images thus obtained were analyzed by SCANCO evaluation software to perform the 3-dimensional structural parameter analysis, including bone volume/tissue volume (BV/TV), bone surface/total tissue volume (BS/TV), trabecular number (Tb.N), trabecular pattern factor (Tb.Pf), trabecular thickness (Tb.Th), and trabecular separation (Tb.Sp).

### Bone biomechanical test

The femur bone samples wrapped in gauze impregnated with normal saline for overnight at 4°C were subjected to biomechanical testing. The 3-point bending load test of femur midshaft was carried out by using a hard tissue testing instrument, Instron 5543, United States, according to the previous studies (Akhter and Jung, 2007). The load and displacement values were recorded, and a curve was plotted. On the basis of the data recorded, the fracture load, fracture displacement, fracture stress, and fracture energy were calculated. These calculated values directly reflect the changes in bone mechanical properties.

### Real time polymerase chain reaction

The total RNA from gastrocnemius was isolated by using RNeasy Mini Kit (Qiagen, Hilden, Germany) after the instruction sheet. The complementary DNA (cDNA) was synthesized by using a High-Capacity cDNA Reverse Transcription Kit (Takara, Japan) in the reverse transcription reaction. The resulting cDNA was used for real-time PCR, performed in an ABI Step One Real-Time PCR System (Applied Biosystems) with Fast SYBR Green Master Mix (Applied Biosystems). Each primer set for the PCR is given in Supplementary Table S1. The thermocycling condition was inactivation of reverse transcriptase at 95°C for 10 s and 60°C for 30 s. The glyceraldehyde-3-phosphate dehydrogenase (GAPDH) was the loading control, and the sample threshold (Ct) values were normalized to GAPDH.

### Histological analysis

The mice gastrocnemii were collected at the end of the study and were fixed with 4% paraformaldehyde in 0.2 M phosphate buffer (pH 7.4) for 24 h at 4°C. The embedded paraffin blocks were placed in a microtome, and 4 µm sections were cut and deparaffinized in xylene for 5 min in a repetition of 3 times. Next, they were rehydrated sequentially in absolute, 99%, 95%, and 80% ethanol for 1 min and counterstained with hematoxylin/eosin. The images of all stained slides were captured under a microscope (E800; Canon, Tokyo, Japan) with a CCD camera. The cross-sectional areas (CSAs) of myofibers were quantified by using ImageJ (NIH ImageJ system, Bethesda, MD, United States).

### Western blot

The samples for western blot were prepared by homogenizing the soleus and gastrocnemius muscles in radioimmunoprecipitation assay buffer supplemented with protease inhibitors, followed by sonication and centrifuged at 15,000 rpm for 20 min at 4°C to obtain the supernatant. The protein concentration in the supernatant was measured by a bicinchoninic acid assay kit (Pierce, Rockford, IL, United States). Equal amounts of protein (30 μg) from each sample were electrophoresed on 8%–12% sodium dodecyl sulfate polyacrylamide gel and transferred onto polyvinylidene difluoride membrane. The membranes were first blocked with non-fat milk (5%) for 1 h at RT before incubating with primary antibodies against GAPDH (1:5000, 60004-1-Ig, Protein tech), Atrogin-1 (1:1000, ab168375, Abcam), MuRF1 (1:1000, ab183094, Abcam), P-PI3K (1:1000, ab182651, Abcam), P-AKT (1:1000, Cell signaling Technology), and PI3K (1:1000, ab191606, Abcam). Membranes were washed with Tris-buffered saline containing 0.1% Tween 20 Detergent, followed by a reaction with respective secondary antibodies for 45–60 min. The immunoblots were developed with enhanced chemiluminescence plus reagent, and the results were quantified with laboratory image version 2.7.1.

### Statistical analysis

Statistical analysis was performed by using SPSS21 software (Chicago, IL, United States). The data are expressed as mean ± standard error of the mean. The *in vitro* experiments were carried out in replicates of a minimum of 3 to reduce error in sampling. The significance of differences between the groups was obtained by applying 1-way ANOVA. The *p* < 0.05 was considered significant.

## Results

### Effect of eldecalcitol on body weight, muscle mass, and strength

**FIGURE 1:**
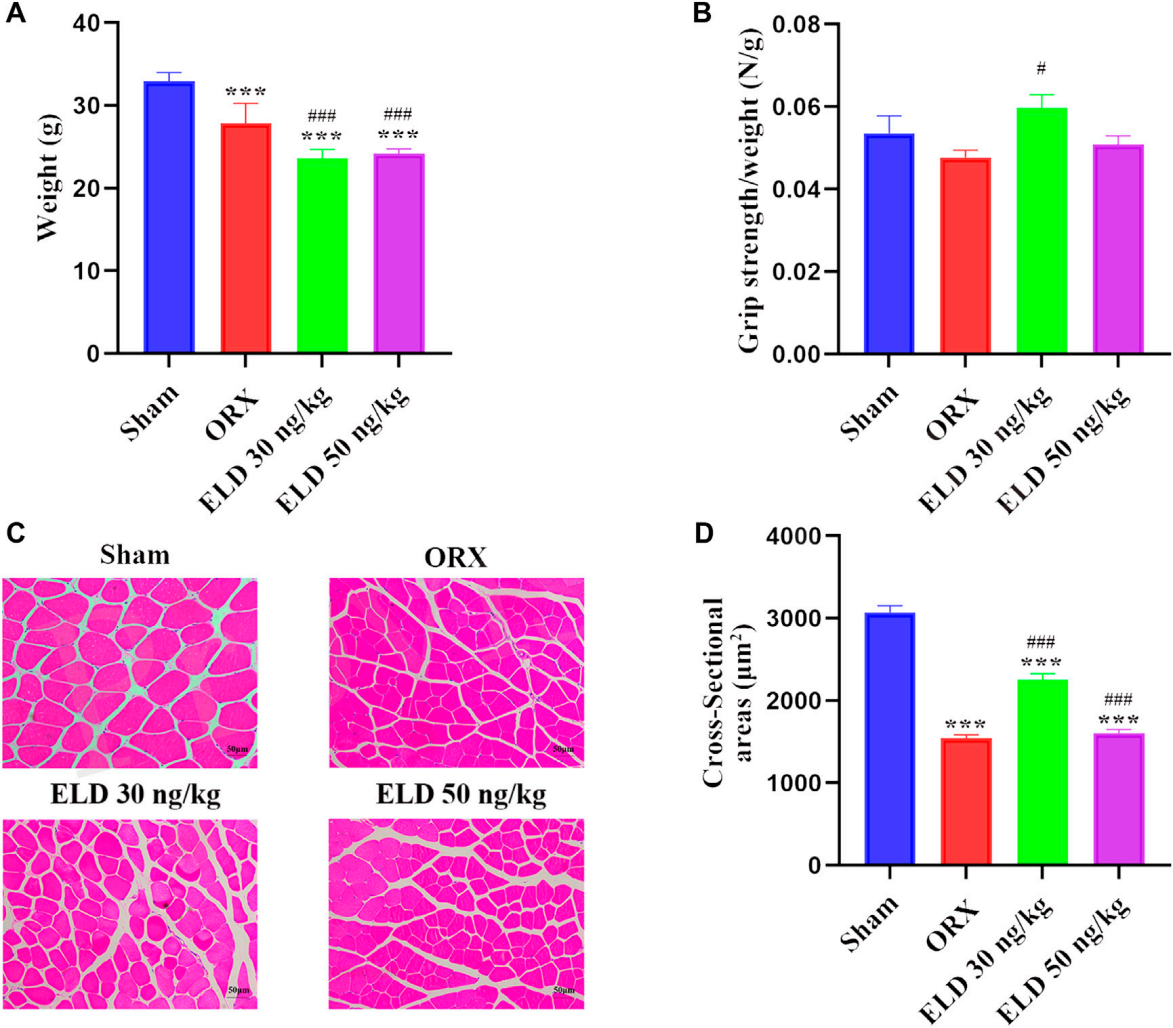
**(A-B)** Body weight and grip strength after 12-week treatment. **(C-D)** H&E staining of gastrocnemius and muscle cross-sectional area of 4 groups. ***p < 0.001 *versus* Sham group; ^#^ *p* < 0.05 *versus* ORX; ^###^ *p* < 0.001 *versus* ORX. Data are expressed as mean ± SEM, *n* > 3. Abbreviations: ELD, eldecalcitol; ORX, orchiectomy; SEM, standard error of mean.

In comparison with the sham mice, body weight was significantly reduced by 18.21% in the ORX mice. In mice treated with eldecalcitol, the body weight reduced compared with ORX although the decrease was similar between the different exposure groups (Figure 1A). Considering the difference between the body weights of mice, the muscle strength was normalized by body weight to better evaluate the muscle strength. The normalized grip strength was higher in animals administered with 30 ng/kg eldecalcitol compared with control (Figure 1B). The grip test showed that 30 ng/kg eldecalcitol completely rescued ORX-induced muscle weakness and increased by 24.53% compared with the ORX group (Figure 1B). The fiber sizes of CSAs in the ORX group decreased by 49.6% compared with the sham group, and those in the eldecalcitol 30 ng/kg group increased by 31.3% compared with the ORX mice (Figures 1C,D).

### Eldecalcitol improved muscle Fiber Type Composition in Mice With ORX-Induced muscle atrophy

**FIGURE 2:**
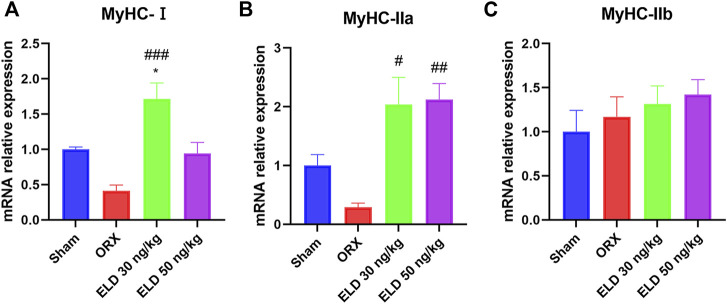
The mRNA level of fiber type composition in mice with ORX-iduced muscle atrophy. **(A)** Slow fiber MyHC-Ⅰ **(B,C)** Fast fiber MyHC-Ⅱa and MyHC-Ⅱb. *p < 0.05 *versus* sham; ^#^ *p* < 0.05 *versus* ORX; ^##^ *p* < 0.01 *versus* ORX; ^###^ *p* < 0.001 *versus* ORX. Data are expressed as mean ± SEM, *n* ≥ 3. Abbreviations: ELD, eldecalcitol; MyHC, Beta-Myosin Heavy Chain; ORX, orchiectomy; SEM, standard error of mean.

The quantitative reverse transcription PCR (RT-qPCR) showed that eldecalcitol enhanced the mRNA levels of type I and IIa fibers compared with the ORX group, and no difference was noted in the expression of type IIb fibers among the groups (Figures 2A–C).

### Effects of Eldecalcitol Administration on ORX-Induced bone loss

**FIGURE 3:**
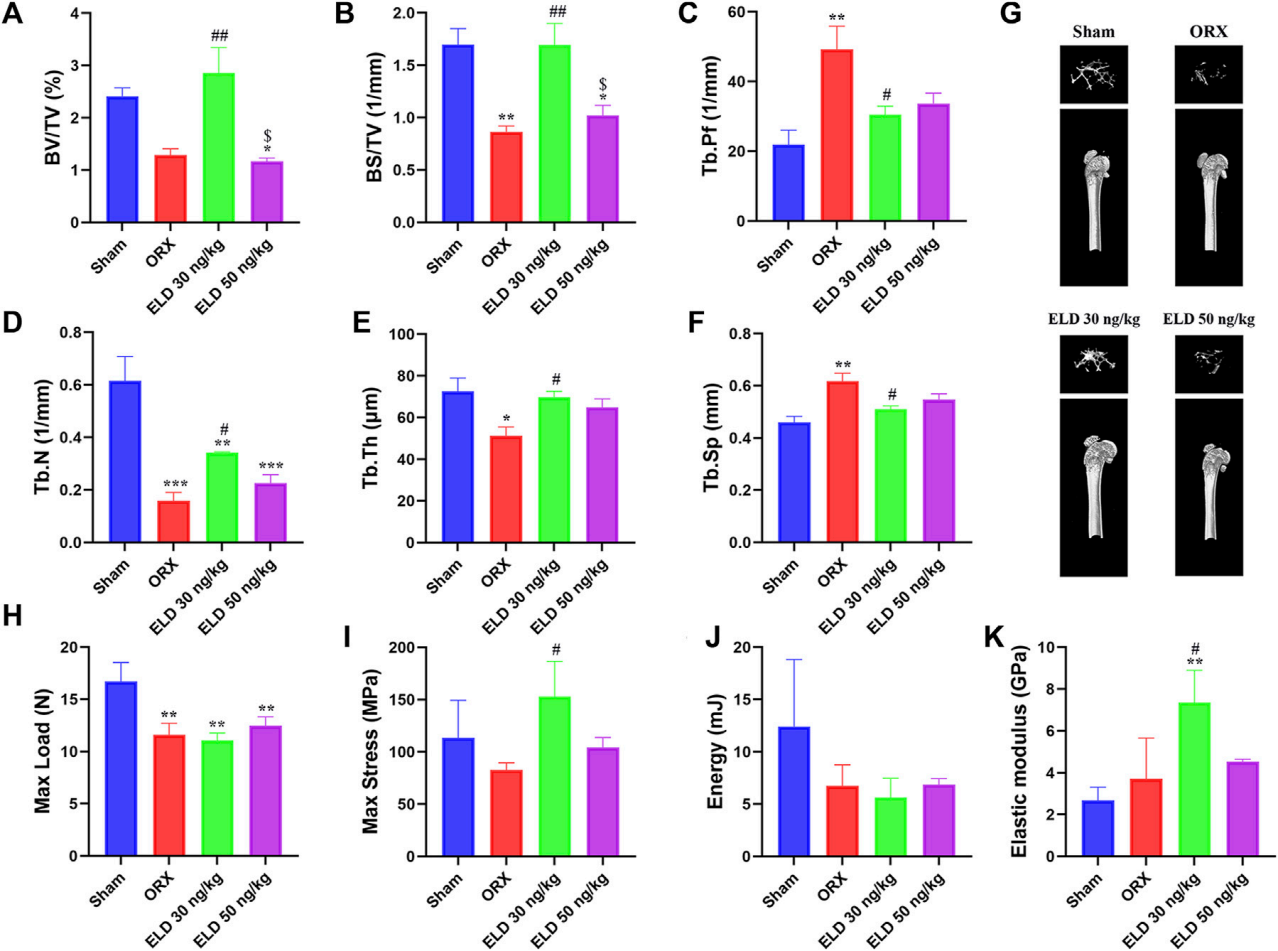
Effect of eldecalcitol administration on ORX-induced bone loss. **(A)** Bone Volume/Total Volume (BV/TV, %) of Distal Femur **(B)** Bone Surface/Total Volume (BS/TV, 1/mm) **(C)** Trabecular Pattern Factor (Tb.Pf, 1/mm) **(D)** Trabecular Number (Tb.N, 1/mm) **(E)** Trabecular Thickness (Tb.Th, μm) **(F)** Trabecular Separation (Tb.Sp, mm) **(G)** Representative Images of Micro-CT of 4 Groups **(H)** Max Load (N) **(I)** Max Stress (MPa) **(J)** Energy (mJ) **(K)** Elastic Modulus (GPa). *p < 0.05 *versus* sham; **p < 0.01 *versus* sham; ***p < 0.001 *versus* sham; ^#^ *p* < 0.05 *versus* ORX; ^##^ *p* < 0.01 *versus* ORX; ^$^ *p* < 0.05 *versus* ELD 30 ng/kg. Data are expressed as mean ± SEM, *n* ≥ 3. Abbreviations: ELD, eldecalcitol; MyHC, Beta-Myosin Heavy Chain; ORX, orchiectomy; SEM, standard error of mean.

After 12 weeks of treatment, the parameters of trabecular bone in the femur were analyzed by using µCT and are shown in Figure 3. Compared with the sham mice, the ORX mice showed reduced bone mass, indicated by 46.62% decrease of BV/TV, 49.05% decrease of BS/TV, 124.72% increase of Tb.Pf, 74.29% decrease of Tb.N, 29.39% decrease of Tb.Th, and 34.44% increase of Tb.Sp (Figures 3A–F). Compared with the mice in the ORX group, 30-ng/kg eldecalcitol dose administration significantly increased the bone mass, with 122% increase of BV/TV, 95.68% increase of BS/TV, 116.12% increase of Tb.N, 36.35% increase of Tb.Th, 17.41% decrease of Tb.Sp, and 38.03% decrease of Tb.Pf compared with the mice in the ORX group (Figures 3A–F). The 3-dimensional image of distal femur with trabecula is shown in Figure 3G. Mice administrated with 30 ng/kg eldecalcitol showed more trabeculae compared with the ORX mice. Interestingly, 30 ng/kg eldecalcitol increased 41.59% of maximum stress and 49.53% of elastic modulus compared with those in the ORX group; however, there were no differences between the 50 ng/kg eldecalcitol and the ORX groups (Figures 3H–K).

### Eldecalcitol decreases the makers of muscle atrophy

**FIGURE 4:**
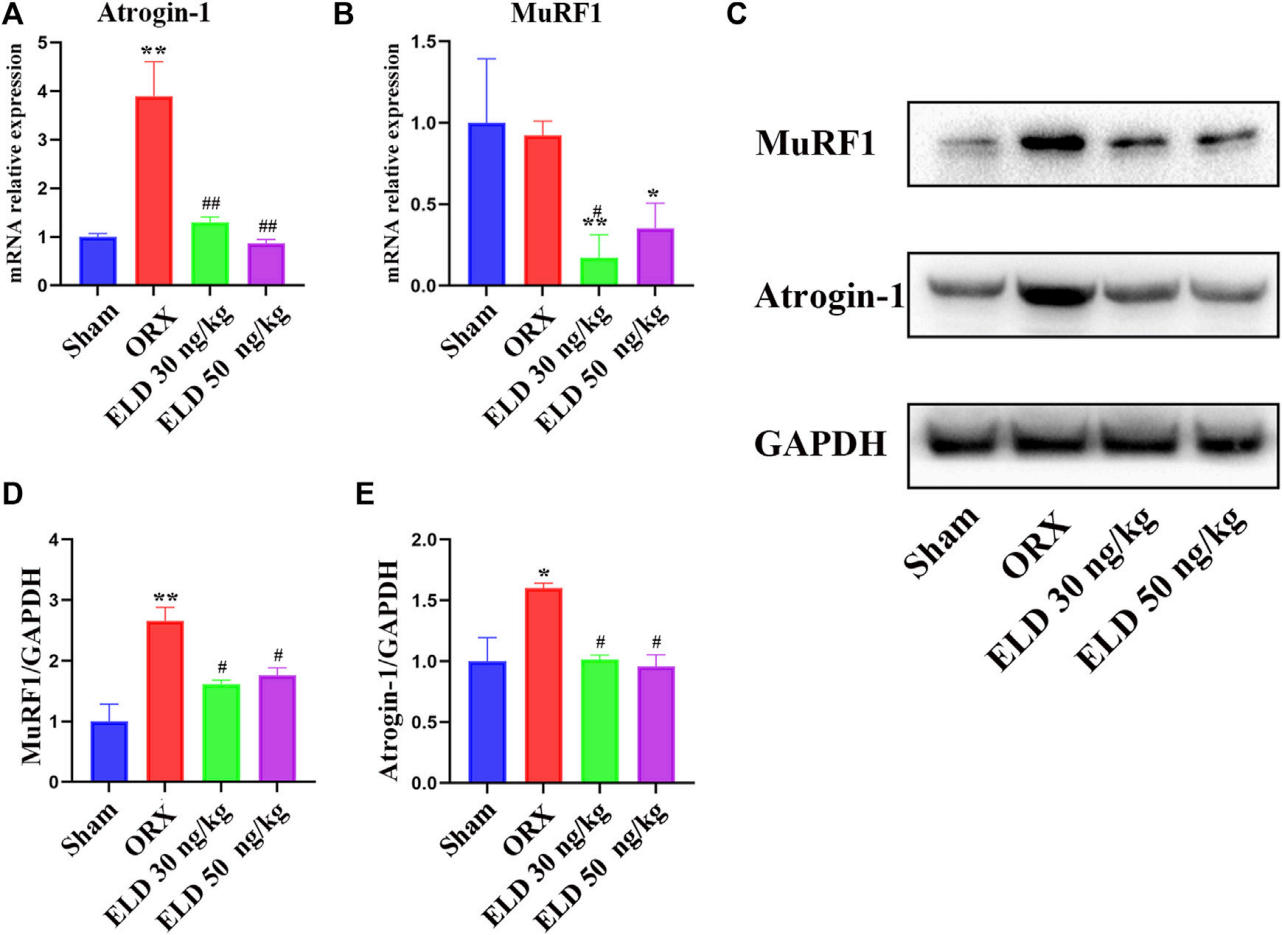
Effects of eldecalcitol on muscle atrophy markers in skeletal muscle of ORX mice **(A-E)** The mRNA level and the protein expression level of muscle atrophy makers Atrogin-1 and MuRF1. *p < 0.05 *versus* sham; **p < 0.01 *versus* sham; ^#^ *p* < 0.05 *versus* ORX; ^##^ *p* < 0.01 *versus* ORX. Data are expressed as mean ± SEM, *n* ≥ 3. Abbreviations: ELD, eldecalcitol; ORX, orchiectomy; SEM, standard error of mean.

The protein or mRNA expression levels of MuRF1 and Atrogin-1 of gastrocnemius in both the 30 ng/kg eldecalcitol and the 50 ng/kg eldecalcitol groups were lower than the Sham group, with significantly lower expression levels observed for mRNA of MuRF1 (Figure 4). The mRNA expression levels of MuRF1 and Atrogin-1 of gastrocnemius in the 30 ng/kg eldecalcitol and the 50 ng/kg eldecalcitol groups were rescued and were much lower compared with the ORX group (Figure 4).

### Eldecalcitol inhibits FOXO transcription factors by activating PI3K/AKT signaling pathways

**FIGURE 5:**
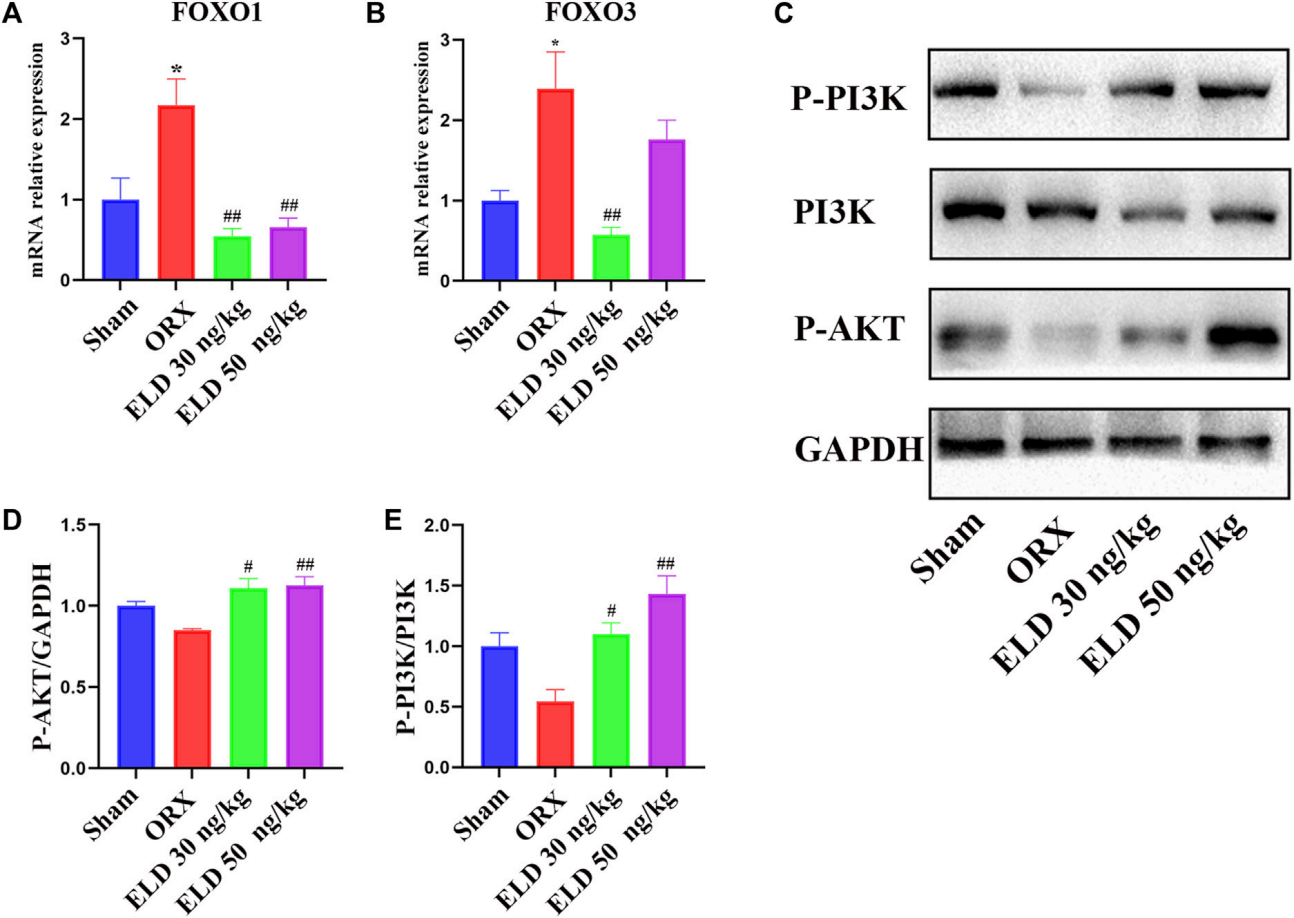
Eldecalcitol exerts its activity by inhibiting FOXO1 and FOXO3 transcription factors and promoting PI3K/AKT signaling pathways. **(A,B)** The mRNA levels of FOXO1nad FOXO3. **(C–E)** The protein levels of P-PI3K, PI3K and P-AKT. *p < 0.05 *versus* sham; **p < 0.01 *versus* sham; ^#^ *p* < 0.05 *versus* ORX; ^##^ *p* < 0.01 *versus* ORX. Data are expressed as mean ± SEM, *n* ≥ 3. Abbreviations: ELD, eldecalcitol; ORX, orchiectomy; GADPH, Glyceraldehyde-3-Phosphate Dehydrogenase; PI3K, Phosphoinositide 3-kinases; P-AKT, phosphorylated AKT; SEM, standard error of mean.

To further investigate the mechanism through which eldecalcitol ameliorated ORX-induced muscle atrophy, proteins in PI3K/AKT/FOXOs signaling pathways were evaluated. After the administration of eldecalcitol in the ORX mice, the mRNA levels of FOXOs and the protein levels of P-PI3K, PI3K, and P-AKT were measured (Figures 5A–E). The ORX mice showed a significant increase in the expression level of transcription factors FOXO1 and FOXO3 at mRNA level, whereas the eldecalcitol treatment reversed these effects (Figures 5A,B). The expression levels of P-PI3K and P-AKT were reduced in the ORX group, whereas eldecalcitol treatment significantly increased the expression levels of P-PI3K and P-AKT (Figures 5C–E). The downstream signaling molecule ratio of P-AKT ameliorated with the recovery of P-PI3K/PI3K and eldecalcitol decreased mRNA expression of FOXOs notably (Figures 5A–E).

All in all, our experiment proves that eldecalcitol improved muscle mass reduction and muscle strength reduction in ORX mice through quantifying the change of cross-sectional areas (CSAs) of myofibers and testing grip strength. Eldecalcitol increased bone mass and promoted bone biomechanical intensity. To clarify the mechanism of eldecalcitol inhibiting muscle atrophy in ORX mice, we detect the levels of phosphorylated PI3K and phosphorylated AKT indicating that PI3K/AKT/FOXOs signaling pathway is activated.

## Discussion

In this study, we examined the effect of the administration of eldecalcitol in the ORX mice. Our findings suggest that both the doses of eldecalcitol decreased body weight in the ORX mice. Furthermore, eldecalcitol was effective in increasing bone mass and strength of femur, preventing muscle atrophy of gastrocnemius in the ORX mice. Furthermore, 30 ng/kg eldecalcitol appeared better in increasing bone strength and recovery of muscle weakness than 50 ng/kg eldecalcitol in the ORX mice. Eldecalcitol 30 ng/kg dose completely rescued ORX-induced bone loss and muscle weakness. Eldecalcitol also enhanced the mRNA levels of type I and IIa fibers in our study, which confirm that eldecalcitol can inhibit the protein degradation in gastrocnemius of ORX mice. Eldecalcitol inhibited FOXO1 and FOXO3 transcription factors and promoted the levels of phosphorylated PI3K and phosphorylated AKT, which suggests that PI3K/AKT/FOXOs signaling pathway is a potential pathway through which eldecalcitol can prevent ORX-induced muscle atrophy.

Previous preclinical studies have shown that eldecalcitol prevents bone loss after ovariectomy by suppressing bone resorption (de Freitas et al., 2011; Harada et al., 2012). Another preclinical study showed that eldecalcitol is a more potent inhibitor of bone resorption than alfacalcidol in an estrogen-deficient rat model of osteoporosis (Uchiyama et al., 2002). A prospective, comparative, randomized trial was conducted in female patients with osteoporosis that demonstrated eldecalcitol has the greater potential to improve cortical and trabecular microstructure at the peripheral bone than alfacalcidol (Ni et al., 2021). Resorption-independent minimodeling was reported in eldecalcitol-treated postmenopausal women, resulting in a greater increase in BMD and bone strength than control (Hikata et al., 2016). In consistent with these published literature, eldecalcitol increased bone mass and promoted biomechanical strength of bone among the ORX mice in our study. Higher BV/TV, BS/TV, Tb.N, Tb.Th and lower Tb.Pf and Tb.Sp were observed in the 30 ng/kg eldecalcitol mice compared with the ORX mice. The biomechanical analysis confirmed that 30 ng/kg eldecalcitol increased the maximum stress, a capacity to withstands loads, and elastic modulus, an indicator of bone toughness (Serizawa et al., 2016; Besschetnova et al., 2019).

Eldecalcitol has role not only in improving bone mass and muscle strength but also in preventing muscle protein degradation and enhancing the mRNA levels of type I and IIa fibers. This was also evident in another study by Saito et al. where they observed increased gene expression of MyHC subtypes IIa, IIb, and IId/x by eldecalcitol (Saito et al., 2017).The type II muscle fibers have features of fast contraction and easy fatigability. Increases in type II muscle fibers are thought to maintain physical balance in momentary postural change, leading to a reduction of falls (Saito et al., 2017). In this study, we confirm again that eldecalcitol has the effect to change the expression of MyHC subtypes.

The proteins levels of MuRF1 and Atrogin-1, the two important E3 ubiquitin ligases, are closely related to skeletal muscle protein catabolism and are the markers of skeletal muscle protein degradation (Hayakawa et al., 2015). A study on C57BL/6J mice suggested that vitamin D deficiency increased the expression levels of MuRF1 and Atrogin-1 protein in the activity-limited gastrocnemius muscle, indicating that vitamin D deficiency resulted in more serious protein degradation of the gastrocnemius muscle during immobilization (Yang et al., 2020). In our study, eldecalcitol inhibited the protein degradation in gastrocnemius muscle of ORX mice.

The precise pharmacological action of the active form of vitamin D on muscle metabolism is not fully understood. It has been shown that c-Fos and RANKL may be the mechanistic targets of vitamin D (Takasu et al., 2006). Meanwhile, the PI3K/AKT pathway plays a key regulation role in myotube hypertrophy and atrophy. AKT also known as protein kinase B (PKB), is a member of the serine/threonine protein kinase AGC family, which comprises three isoforms in mammalian cells, AKT1/PKBα, AKT2/PKBβ, and AKT3/PKBγ, and plays a critical role in all kinds of pathogenic pathways. An increase in AKT level during hypertrophy and a decrease in AKT level during atrophy, has been demonstrated in mice (Szewczyk et al., 2007). Besides, AKT has been found to produce a marked effect in muscle atrophy caused by a variety of pathogeny including diabetes type II-related sarcopenia, muscle atrophy induced by cachexia and glucocorticoid (Cetrone et al., 2014; Meng et al., 2022; Mishra et al., 2022). In addition, AKT involves in the regulation of different effectors, including ion channels that regulate cell proliferation in cell lines and skeletal muscle (Cetrone et al., 2014; Maqoud et al., 2018). Previous studies have shown that FOXO1 in skeletal muscles plays an important role in muscle atrophy. For instance, muscle atrophy was associated with the overexpression of FOXO1 gene in transgenic mice, and FOXO1 has already been shown to negatively regulate type I fibers (Kamei et al., 2004). FOXO1 also activates the expression of atrophy-related genes such as Atrogin 1 and cathepsin L in various muscle atrophy-related conditions (Yamazaki et al., 2010; Kamei et al., 2014). Active FOXO3 also causes dramatic atrophy by acting on the Atrogin 1 promoter (Sandri et al., 2004). In a previous study, vitamin D suppressed the glucocorticoid-induced gene expression of Atrogin 1 and cathepsin L in C2C12 myoblasts *via* the FOXO1-mediated pathway in muscle cells (Hirose et al., 2018). Similarly in our study, eldecalcitol decreased FOXO1 and FOXO3 and promoted phosphorylation of AKT and PI3K.

A dose-dependent study with eldecalcitol at doses of 0.05, 0.1, and 0.2 μg/kg showed gradual improvements in BMD and mechanical properties in an ovariectomized rat model (Uchiyama et al., 2002). On the basis of this model, we used eldecalcitol doses in mice ranging from 30 to 50 ng/kg, both 3 days per week, which was between 1.0-fold and 1.7-fold higher than the 0.75 μg daily dose administered to a human weighing 60 kg. In Japan, eldecalcitol has been used in clinical trial to treat type II osteoporosis at a daily dose of 0.5, 0.75, or 1.0 μg/d, and the results demonstrate that eldecalcitol treatment at around 0.75 μg/d can effectively and safely increase lumbar and hip BMD in patients with osteoporosis (Matsumoto et al., 2011; Hagino, 2013), which was consistent with our study that showed a 30 ng/kg eldecalcitol dose was better than 50 ng/kg dose and had better effect on bone mass, muscle mass, and promoted muscle strength. Eldecalcitol has been approved for treating osteoporosis in Japan since 2011. In recent studies, eldecalcitol was found to be associated with significantly increased urine calcium. Hypercalcemia, acute kidney injury (AKI), and urolithiasis (Takeuchi et al., 2022). It may be because it can reduce the resorption of blood calcium, so that the urinary calcium level is relative elevated. Vitamin D supplements also present the same confusion. Some studies have also pointed out that vitamin D increases hypercalciuria (Aloia et al., 2018). On the contrary, It is also reported that the increase of urinary calcium is not associated with increased serum vitamin D and may result from other factors, such as dietary factors (Taheri et al., 2019). Therefore, more clinical studies need to clarify the adverse reactions of eldecalcitol in different populations.

## Conclusion

Our study is the first to delineate the role of eldecalcitol in improving bone mass and strength, inhibiting muscle atrophy, and promoting muscle fiber transition in orchiectomized mice. Eldecalcitol reduces bone loss in ORX mice and acts on muscle atrophy by activating PI3K/AKT/FOXOs pathways. Future research on other pathways for the potential mechanism of eldecalcitol remains to be elucidated.

## Data Availability

The original contributions presented in the study are included in the article/Supplementary Material, further inquiries can be directed to the corresponding author.
