# Supplementary material for: Eldecalcitol prevents muscle loss by suppressing PI3K/AKT/FOXOs pathway in orchiectomized mice
Source: Front Pharmacol. 2022 Oct 28;13:1018480. doi: 10.3389/fphar.2022.1018480 (PMC9650589; doi:10.3389/fphar.2022.1018480)
Supplement: Supplementary file 1 [file Table1.docx]

**Supplementary Table S1.** Sequences of Primers Used for Reverse Transcription‑Quantitative PCR

| mMyHC I-F | GCACTACAGTGGCGACTCAGATGC |
| --- | --- |
| mMyHC I-R | CACTGTAGTCGGTGTCGTAGCC |
| mMyHC IIb-F | AGGACCAACTGAGTGAAGTGA |
| mMyHC IIb-R | GGGAAAACTCGCCTGACTCTG |
| mMyHC IIa-F | TAAACGCAAGTGCCATTCCTG |
| mMyHC IIa-R | GGGTCCGGGTAAGCTGG |
| FOXO1-F | GCGGGCTGGAAGAATTCAAT |
| FOXO1-R | TCCAGTTCCTTCATTCTGCA |
| Atrogin-1-F | GCAGAGAGTCGGCAAGTC |
| Atrogin-1-R | CAGGTCGGTGATCGTGAG |
| Murf1-F | CAACCTGTGCCGC-AAGTG |
| Murf1-R | CAACCTCGTGCCTACAAGATG |
| FOXO3-F | ACAAACGGCTCACTTTGTCCCAGA |
| FOXO3-R | TCTTGCCCGTGCCTTCATTCT |

F, forward; R, reverse
